# A High Diversity of Eurasian Lineage Low Pathogenicity Avian Influenza A Viruses Circulate among Wild Birds Sampled in Egypt

**DOI:** 10.1371/journal.pone.0068522

**Published:** 2013-07-12

**Authors:** Nancy A. Gerloff, Joyce Jones, Natosha Simpson, Amanda Balish, Maha Adel ElBadry, Verina Baghat, Ivan Rusev, Cecilia C. de Mattos, Carlos A. de Mattos, Luay Elsayed Ahmed Zonkle, Zoltan Kis, C. Todd Davis, Sam Yingst, Claire Cornelius, Atef Soliman, Emad Mohareb, Alexander Klimov, Ruben O. Donis

**Affiliations:** 1 Influenza Division, Centers for Disease Control and Prevention, Atlanta, Georgia, United States of America; 2 National Center for Epidemiology, Budapest, Hungary; 3 Viral and Zoonotic Diseases Research Program, United States Naval Medical Research Unit 3, Cairo, Egypt; 4 Antiplague Institute, Odessa, Ukraine; 5 Ministry of Environment, Cairo, Egypt; University of Georgia, United States of America

## Abstract

Surveillance for influenza A viruses in wild birds has increased substantially as part of efforts to control the global movement of highly pathogenic avian influenza A (H5N1) virus. Studies conducted in Egypt from 2003 to 2007 to monitor birds for H5N1 identified multiple subtypes of low pathogenicity avian influenza A viruses isolated primarily from migratory waterfowl collected in the Nile Delta. Phylogenetic analysis of 28 viral genomes was performed to estimate their nearest ancestors and identify possible reassortants. Migratory flyway patterns were included in the analysis to assess gene flow between overlapping flyways. Overall, the viruses were most closely related to Eurasian, African and/or Central Asian lineage low pathogenicity viruses and belonged to 15 different subtypes. A subset of the internal genes seemed to originate from specific flyways (Black Sea-Mediterranean, East African-West Asian). The remaining genes were derived from a mixture of viruses broadly distributed across as many as 4 different flyways suggesting the importance of the Nile Delta for virus dispersal. Molecular clock date estimates suggested that the time to the nearest common ancestor of all viruses analyzed ranged from 5 to 10 years, indicating frequent genetic exchange with viruses sampled elsewhere. The intersection of multiple migratory bird flyways and the resulting diversity of influenza virus gene lineages in the Nile Delta create conditions favoring reassortment, as evident from the gene constellations identified by this study. In conclusion, we present for the first time a comprehensive phylogenetic analysis of full genome sequences from low pathogenic avian influenza viruses circulating in Egypt, underscoring the significance of the region for viral reassortment and the potential emergence of novel avian influenza A viruses, as well as representing a highly diverse influenza A virus gene pool that merits continued monitoring.

## Introduction

Avian influenza viruses (AIV) are ubiquitous and their natural reservoir is believed to be aquatic birds in which a majority of subtypes has been identified [1], [2]. Subtype H5 and H7 AIV circulating in wild birds can become highly pathogenic in poultry populations resulting in significant morbidity and mortality and, as is the case for the currently circulating highly pathogenic influenza A (H5N1) [HPAI H5N1]virus, sporadic human infection with significant mortality rates [3], [4], [5], [6], [7]. Furthermore, AIV pose a threat for human and animal health as new variants could emerge leading to pandemics or epizootics. Recently, human infections with avian derived low pathogenic avian influenza (LPAI) H10N7 virus were reported from Australia [8] and a human infection with an avian influenza A (H9N2) virus was described in Bangladesh [9]. Thus, risk assessment that begins by monitoring the circulation and gene flow of AIV in their natural hosts is crucial for pandemic preparedness [10].

The Nile Delta of northern Egypt is one of the world’s most important bird migration routes and serves as a vital stopover for millions of birds making their annual migration between the Palearctic and Afrotropical regions [11]. Two major migratory flyways, the Black Sea-Mediterranean and East African-West Asian flyway, overlap in Egypt [12], [13], [14]. This region is a wintering ground for hundreds of thousands of aquatic birds that host influenza A viruses; e.g. species of ducks, gulls, and shorebirds known to harbor LPAI viruses and, in rare occasions, HPAI H5N1 viruses [1]. In fact, the first evidence of HPAI H5N1 in Egypt was the detection of viral RNA in a common teal (*Anas crecca*) captured in the Nile Delta region of Damietta in December 2005 [15]. In addition, the first reports of H5N1 in Egyptian poultry noted the high sequence similarity with viruses detected in wild birds in Europe at that time period and suggested that migratory birds introduced the virus into the country [16]. In order to monitor the wild bird population for infection with HPAI H5N1, the US Naval Medical Research Unit No. 3 (Cairo, Egypt), in collaboration with the Egyptian Ministry of Environment, had initiated sampling in several sites in 2003 when H5N1 started to spread from China into other countries [17].

Although many African countries have reported outbreaks of HPAI H5N1, they have usually been sporadic with limited sustained spread [18], [19]. In contrast, Egypt has experienced frequent outbreaks of HPAI H5N1 from many different regional governorates since it was first introduced and due to the continuous circulation of the virus in poultry, surveillance in wild birds to monitor re-introduction and/or spread has been a priority [18]. While the large majority of influenza A virus positive samples were found to be H5N1 negative (>99%; data not shown), this study sought to determine the subtypes, nearest common ancestors and extent of genetic diversity of the LPAI viruses that were collected from this surveillance. LPAI viruses collected from migratory waterfowl sampled over five consecutive years (2003–2007) in four different regions of Egypt were analyzed and complete genome sequences of 28 viruses were generated and characterized to better understand the population genetics of LPAI viruses detected in and around the Nile Delta and to describe influenza A virus gene flow among different subtypes potentially originating from multiple migratory bird flyways.

## Materials and Methods

### Ethics Statement

Bird capture, collection and sampling methods were reviewed and approved by the US Naval Medical Research Unit Number 3 Institutional Animal Care and Use Committee (IACUC 04-01). The animals sampled for this study did not include endangered or protected species. The sampling of wild birds was approved by the Ministry of State for Environmental Affairs in Egypt and carried out on public land.

### Wild Bird Surveillance and Sample Collection

Wild birds were sampled between December 2003 and February 2007 at 12 collection sites in Egypt; 9 located within the Nile Delta, 1 on the Sinai Peninsula (Port Said), 1 in the southeast of the country near Aswan and 1 south of Lake Nasser near Abu Simbel. Oropharyngeal and/or cloacal specimens were collected with dacron swabs from birds trapped using mist nets and restrained without anesthesia or of from hunter-killed bird carcasses [20]. Twenty-nine different avian species were sampled including birds from the orders Anseriformes, Charadriiformes, Gruiformes, Ciconiiformes, Pelecaniformes, Falconiformes, and Columbiformes; although waterfowl species such as pintail (*Anas acuta*), teal, and shoveler (*Anas clypeata*) ducks were the most frequently sampled.

### Virus Isolation, Subtype Detection and Full Genome Sequencing

Original specimens were screened for influenza A virus using a real-time reverse transcription (RT)-PCR detection kit targeting the matrix (M) gene as previously described [21]. Positive samples were inoculated into 10–11 day old embryonated chicken eggs (ECEs) and allantoic fluid was harvested 24 to 48 hours post-inoculation and screened for the presence of virus by a hemagglutination assay using turkey red blood cells. All infectious materials were maintained in biosafety level 3 containment, including enhancements required by the U.S. Department of Agriculture and the Select Agents program (http://www.cdc.gov/od/ohs/biosfty/bmbl5/bmbl5toc.htm). Genomic RNA was extracted from virus-infected allantoic fluid using the RNeasy extraction kit (Qiagen, Valencia, CA) and used as template for generation of cDNA by random hexamer-primed reverse transcription. Subtyping was performed using a multiplex PCR assay designed to target conserved regions of each of the 16 hemagglutinin (HA) genes and 9 neuraminidase (NA) genes. Forward sense, M13-tagged degenerate primers were designed for all 16 HAs and 9 NA genes and were paired with reverse sense, M13-tagged HA and NA primers targeting conserved regions of the 3′UTR of the minus-sense genomic RNA (Table S1). In each multiplex PCR assay at least 3 forward primers were combined with the HA or NA gene reverse primer (primer combinations available upon request). The PCR product was visualized by gel electrophoresis and amplicons were sequenced using M13 forward and reverse primers. Sequences of HA and NA fragments generated were analyzed using *basic local alignment search tool* (BLAST; available from http://blast.ncbi.nlm.nih.gov/Blast.cgi) against the GenBank database to predict the influenza A subtype. The surface and internal protein genes were then amplified using influenza A virus specific primers as overlapping fragments with the Access Quick one-step RT-PCR kit (Promega, Madison, WI) and subsequently sequenced on an automated Applied Biosystems 3730 system using cycle sequencing dye terminator chemistry (Life Technologies, Carlsbad, CA). Contigs of full length open reading frames were generated for each gene (Sequencher 4.8, Gene Codes, Ann Arbor, MI). Gene sequences were submitted to GISAID (http://platform.gisaid.org) prior to publication (Accession Nos.: EPI 120183–120208, 120210, 120216).

### Dataset Preparation, Phylogenetic Analysis and Molecular Characterization

The presence of multibasic cleavage sites or other insertions at the cleavage site of the HA0 was determined by comparing the coding region of the HA0 protein of each virus to known LPAI viruses. For HA and NA subtype-specific phylogenetic comparison, individual datasets contained a representative selection of publicly available sequences and neighbor-joining (NJ) trees were calculated to identify closest ancestors (Figures S1, S2). All HA and NA gene sequences of Egyptian LPAI viruses sequenced for this study were then aligned to reference sequences of subtypes HA H1 to H16 and NA N1 to N9 containing the nearest ancestors identified. HA and NA NJ trees were then calculated and visualized. For characterization of the internal gene segments, large datasets of polymerase basic protein 2 (PB2; n = 2501), polymerase basic protein 1 (PB1; n = 2380), polymerase acid protein (PA; n = 2142), nucleoprotein (NP; n = 2001), matrix protein (MP; n = 4190) and nonstructural protein (NS; n = 4585) gene sequences were aligned. Included in the datasets were 21 genomes of African LPAI viruses (Zambia, n = 13; Egypt, n = 1; Nigeria, n = 3; South Africa, n = 4) and 6 genomes of LPAI viruses from Ukrainian migratory birds that were sequenced during the course of this study (Accession Nos.: EPI 120209, 120211–120215). To identify possible clusters with recent HPAI H5N1 viruses, publicly available H5N1 sequences were included in datasets and annotated as described previously [22]. Sequence alignments were implemented in BioEdit and calculated with the MUSCLE algorithm [23], [24]. Alignments were manually edited for frameshifts and sequence duplication and only sequences with at least 90% of the coding region were included. Trees were inferred using the NJ method with a Kimura 2-parameter implemented in MEGA version 4 [25].

### Bayesian Analysis and Estimation of the Time to the Most Recent Common Ancestor (TMRCA)

Reduced datasets contained 92 viral sequences with the same viruses for each of the six internal gene segments included when possible. The PB2 gene sequence was missing for virus A/shoveler/Egypt/14029/2006 (H1N1) due to insufficient genomic material. Attempts to re-passage the virus were unsuccessful. Selection criteria were based on subtype, geographic origin, date of collection, and genetic distance to viruses analyzed in this study determined from previous large NJ trees. To calculate time to the most recent common ancestor (TMRCA), the date of collection (day/month/year) for each individual virus in the dataset was transformed into numerical values. The middle of the collection year was used for viruses without precise collection dates. The BEAST software package version 1.6.1 was used for TMRCA analyses [26], [27]. Convergence of data was evaluated with the software Tracer version 1.5 after removal of 10% burn-in [28]. The analysis was repeated with modified parameters and adjusted mean substitutions rates until the effective sample size (ESS) of each prior was at least 200. The model that best fit our data best was the SRD06 codon partition model using the HKY nucleotide substitution model for each partition with unlinked frequencies. The molecular clock model used was an uncorrelated lognormal relaxed clock with an estimated rate of 1. The tree priors were coalescent Bayesian Skyline with 10 groups and piecewise constant skyline model with a randomly generated tree as a start. Each Markov chain Monte Carlo (MCMC) run had a chain length of 100 million and was sampled every 1000 generations. For each TMRCA a credible interval (Bayesian confidence interval) is given as the highest posterior density (HPD 95%) that represents an interval in the domain of a posterior probability distribution. Maximum clade credibility (MCC) trees were generated in TreeAnnotator [26] with 10% burn-in removed, posterior probabilities on 0.5, and median heights. Trees were visualized in FigTree version 1.3.1 and posterior probabilities are shown on or above nodes [29].

In order to infer the geographic dispersal of each virus in the dataset, one of the major flyways was assigned according to the geographical location of that individual virus’s collection site; Black Sea-Mediterranean (BS-MED), East African-West Asian (EA-WA), Central Asian (CA), and the East Asian-Australian (EA-AUS) flyway [12], [30]. Egyptian viruses that did not cluster with other viruses of known geographic collection location were not assigned to a specific flyway due to the overlap of the BS-MED and EA-WA flyway at collection sites and are instead referred to as “Nile Delta stopover”. Each gene cluster in the MCC tree with a high posterior probability (≥0.7) were grouped and classified as belonging to one of the major flyways. Clusters were designated as belonging to a “mixed” flyway group when viruses of different geographical collection location formed a monophyletic cluster. Single viruses that did not group with any of the other viruses in the small phylogenetic trees were assigned to flyways based on their clusters in big phylogenetic trees (data not shown).

## Results

### Surveillance, Virus Epidemiology and Subtype Distribution

Influenza A virus was detected in 731 of the 7678 specimens (9.5%) tested in the five-year interval between 2003 and 2007 (Table 1). A subset of 33 specimen were available for virus isolation. In total 28 LPAI viruses were isolated from wild birds of the order Anseriformes species; Northern shoveler (n = 17, *Anas clypeata*), Common teal (n = 10, *Anas crecca*) and Egyptian goose (n = 1, *Alopochen aegyptiacus*) (Table 1). Viruses were collected in the Damietta Governorate located in the northern Nile Delta region (n = 24), the Port Said Governorate (n = 1), the Aswan Governorate (n = 1), and Abu Simbel located at Lake Nasser in southern Egypt (n = 2) (Table 2). We found 8 different hemagglutinin (HA) subtypes and 8 different neuraminidase (NA) subtypes accounting for 15 unique subtypes (Table 2). The predominate subtypes for HA were H10 (n = 9) and H7 (n = 8) and for NA, N7 (n = 8) and N1 (n = 7) (Table 2).

**Table 1:** Number of influenza A virus PCR positive specimen and number of embryonated chicken egg isolates.

	Bird species(*Latin name*)	Number
Total specimen collected		7678
PCR positive specimen		731
Isolation attempted		33
Isolated viruses		28
	Northern shoveler(*Anas clypteata*)	17
	Common teal(*Anas crecca*)	10
	Egyptian goose(*Alopochen aegyptiacus*)	1
Isolation negative		5

**Table 2:** Avian influenza viruses analyzed in this study with subtype, collection site (Governorate in Egypt), date of collection (DOC) and amino acid sequence of the cleavage site (*) of the HA0 protein.

Virus	Subtype	Governorate	DOC	Cleavage site
A/teal/Egypt/00677-NAMRU3/2004	H1N1	Damiatta	1/28/2004	PSIQS–-R*G
A/shoveler/Egypt/00134-NAMRU3/2005	H1N1	Damiatta	1/13/2005	PSIQS–-R*G
A/shoveler/Egypt/14029-NAMRU3/2006	H1N1	Damiatta	12/8/2006	PSIQS–-R*G
A/teal/Egypt/01351-NAMRU3/2007	H1N1	Damiatta	1/26/2007	PSIQS–-R*G
A/teal/Egypt/20431-NAMRU3/2003	H1N2	Damiatta	12/22/2003	PSIQS–-R*G
A/teal/Egypt/09888-NAMRU3/2005	H4N6	Damiatta	10/3/2005	PEKAS–-R*G
A/shoveler/Egypt/20313-NAMRU3/2003	H5N2	Damiatta	12/15/2003	PRE––TR*G
A/shoveler/Egypt/13251-NAMRU3/2006	H6N2	Damiatta	12/2/2006	PQIET–-R*G
A/teal/Egypt/13203-NAMRU3/2006	H6N2	Damiatta	12/2/2006	PQIET–-R*G
A/shoveler/Egypt/14879-NAMRU3/2006	H7N1	Damiatta	12/22/2006	PELPK–GR*G
A/shoveler/Egypt/00597-NAMRU3/2004	H7N1	Damiatta	1/27/2004	PEIPK–GR*G
A/shoveler/Egypt/00017-NAMRU3/2007	H7N3	Damiatta	12/29/2006	PEIPK–GR*G
A/shoveler/Egypt/00241-NAMRU3/2007	H7N3	Damiatta	1/5/2007	PEIPK–GR*G
A/teal/Egypt/00835-NAMRU3/2004	H7N7	Damiatta	2/18/2004	PEIPK–GR*G
A/shoveler/Egypt/09864-NAMRU3/2004	H7N7	Damiatta	12/22/2004	PEIPK–GR*G
A/Egyptian goose/Egypt/05588-NAMRU3/2006	H7N7	Aswan	4/7/2006	PEIPK–GR*G
A/shoveler/Egypt/00215-NAMRU3/2007	H7N9	Damiatta	1/5/2007	PEIPK–GR*G
A/teal/Egypt/12908-NAMRU3/2005	H10N1	Port Said	11/21/2005	PEIMQ–GR*G
A/shoveler/Egypt/00006-NAMRU3/2007	H10N1	Damiatta	12/29/2006	PEIMQ–GR*G
A/shoveler/Egypt/01574-NAMRU3/2007	H10N4	Damiatta	2/9/2007	PEIMQ–GR*G
A/shoveler/Egypt/00600-NAMRU3/2004	H10N7	Damiatta	1/27/2004	PEIMQ–GR*G
A/shoveler/Egypt/09782-NAMRU3/2004	H10N7	Abu Simbel	12/18/2004	PEIMQ–GR*G
A/shoveler/Egypt/09781-NAMRU3/2004	H10N7	Abu Simbel	12/18/2004	PEIMQ–GR*G
A/shoveler/Egypt/01198-NAMRU3/2007	H10N7	Damiatta	1/19/2007	PEIMQ–GR*G
A/teal/Egypt/01207-NAMRU3/2007	H10N7	Damiatta	1/19/2007	PEIMQ–GR*G
A/shoveler/Egypt/00004-NAMRU3/2007	H10N9	Damiatta	12/29/2006	PEIMQ–GR*G
A/teal/Egypt/00688-NAMRU3/2004	H11N9	Damiatta	1/28/2004	PAIAS–-R*G
A/teal/Egypt/11974-NAMRU3/2005	H13N8	Damiatta	9/10/2005	PAISN–-R*G

### Phylogenetic Relationships of the HA and NA Genes

**Figure 1:**
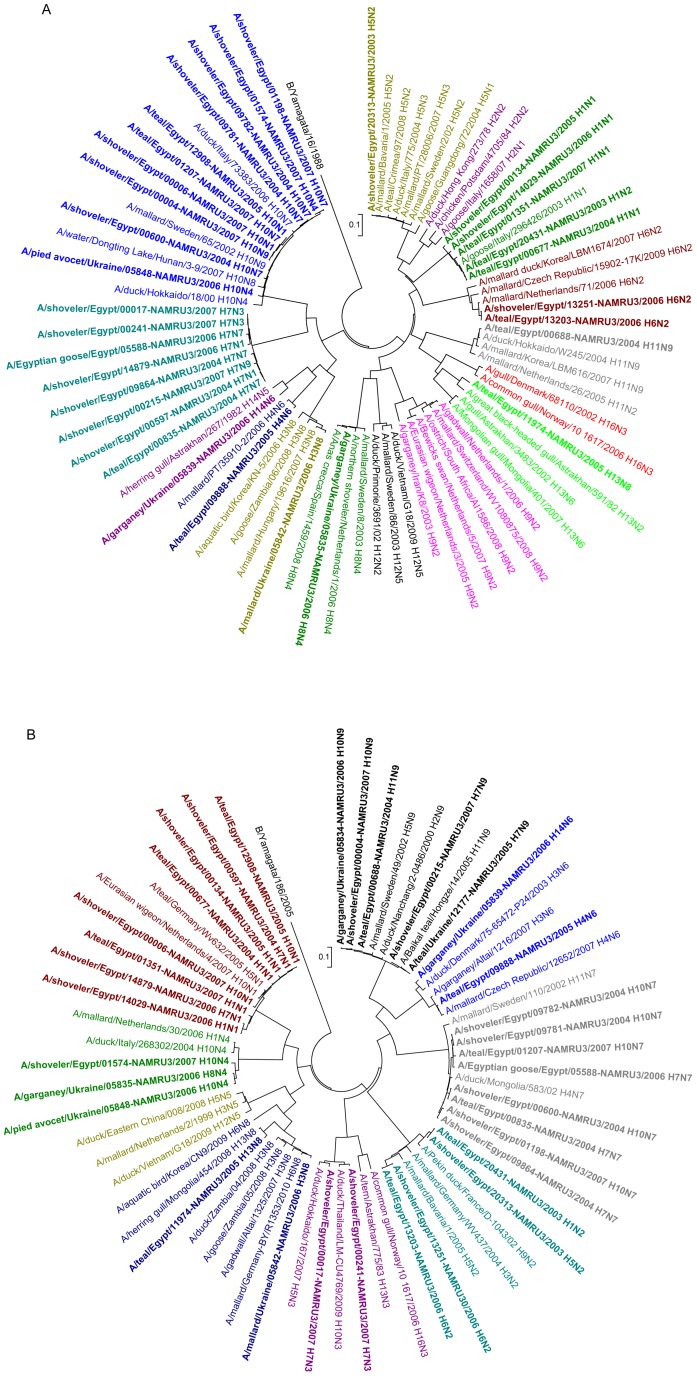
Phylogeny of HA gene sequences of H1 to H16 subtypes (A) and NA gene sequences of N1 to N9 subtypes (B). Viruses identified in this study from Egypt and Ukraine are in boldface. NJ trees were calculated with Mega4 and color coded for each subtype [25]. For Figure 1A H2, H12, H15, H16 HA sequences and for Figure 1B N5 NA gene sequences were included as reference only. Trees are rooted on an influenza B virus sequence (B/Yamagata/186/2005).

All HA and NA gene sequences of Egyptian viruses grouped according to their specific subtype in phylogenetic trees (Figure 1). When analyzed individually, HA gene segments of different subtypes appeared to have evolved closely with European or Eurasian viruses also collected from wild bird species (Figure S1). New World viruses clustered in separate groups for all subtypes in both genes. Both H5 and H7 HA phylogenies indicated a nearest ancestry with recent AIV from wild birds or poultry from Europe (Spain, Sweden, and Netherlands) and Central Asia (Mongolia), while H5 and H7 viruses with highly pathogenic cleavage sites formed separate groups (Figure S1). H10 HA genes from all Egyptian viruses formed a discrete cluster except for A/shoveler/Egypt/00600-NAMRU3/2004 subtype (H10N7) that grouped close to A/duck/Mongolia/149/03 (H10N5) indicating a different genetic origin. Unlike other subtypes, the H11 virus sequence, A/teal/Egypt/00688-NAMRU3/2004 (H11N9), grouped in a mixed cluster with viruses from Europe, Africa, Asia, and Oceania within the same subtype (except for one virus) (Figure S1). None of the viruses sequenced possessed a polybasic cleavage site indicative of a highly pathogenic phenotype (Table 2).

NA phylogenies revealed that Egyptian viruses shared common ancestors with AIV from Europe (Germany, Netherlands, Sweden) and/or Eurasia (Mongolia, Far East Russia) (Figure S2). Independent of their subtypes, N1 neuraminidase genes (H1N1, H10N1, H7N1) from this study clustered together and were distinct from the HPAI H5N1 AIV genes (Figure S2). The N7 sequences grouped in 3 defined clusters, with one group closely related to European viruses and two clusters with viruses from Southeast Asia (Japan) and Central Asia (Mongolia) suggesting multiple independent sources (Figure S2). Unlike other subtypes, the N8 sequence of A/teal/Egypt/11974-NAMRU3/2005 (H13N8) was closely related to recent H13N8 viruses that formed a separate cluster from all other HxN8 AIV in the phylogenetic tree (Figure S2). In the N2 phylogeny, H5N2 and H1N2 viruses grouped together with European viruses, whereas H6N2 viruses clustered together with other European viruses but all shared a common Asian ancestry with A/duck/Nanchang/1749/1992 (H11N2). The H7N3 NA sequences were separated with one virus grouping with European and Asian viruses, whereas A/shoveler/Egypt/00017-NAMRU3/2007 (H7N3) was more closely related to Asian viruses (Figure S2). Similarly, the N4 sequence (only 42 sequences available), the N6 sequence and the three N9 (72) sequences, shared common ancestors predominantly with European or Central Asian AIV (Figure S2).

### Phylogeny of Internal Genes (PB2, PB1, PA, NP, M, NS)

**Figure 2:**
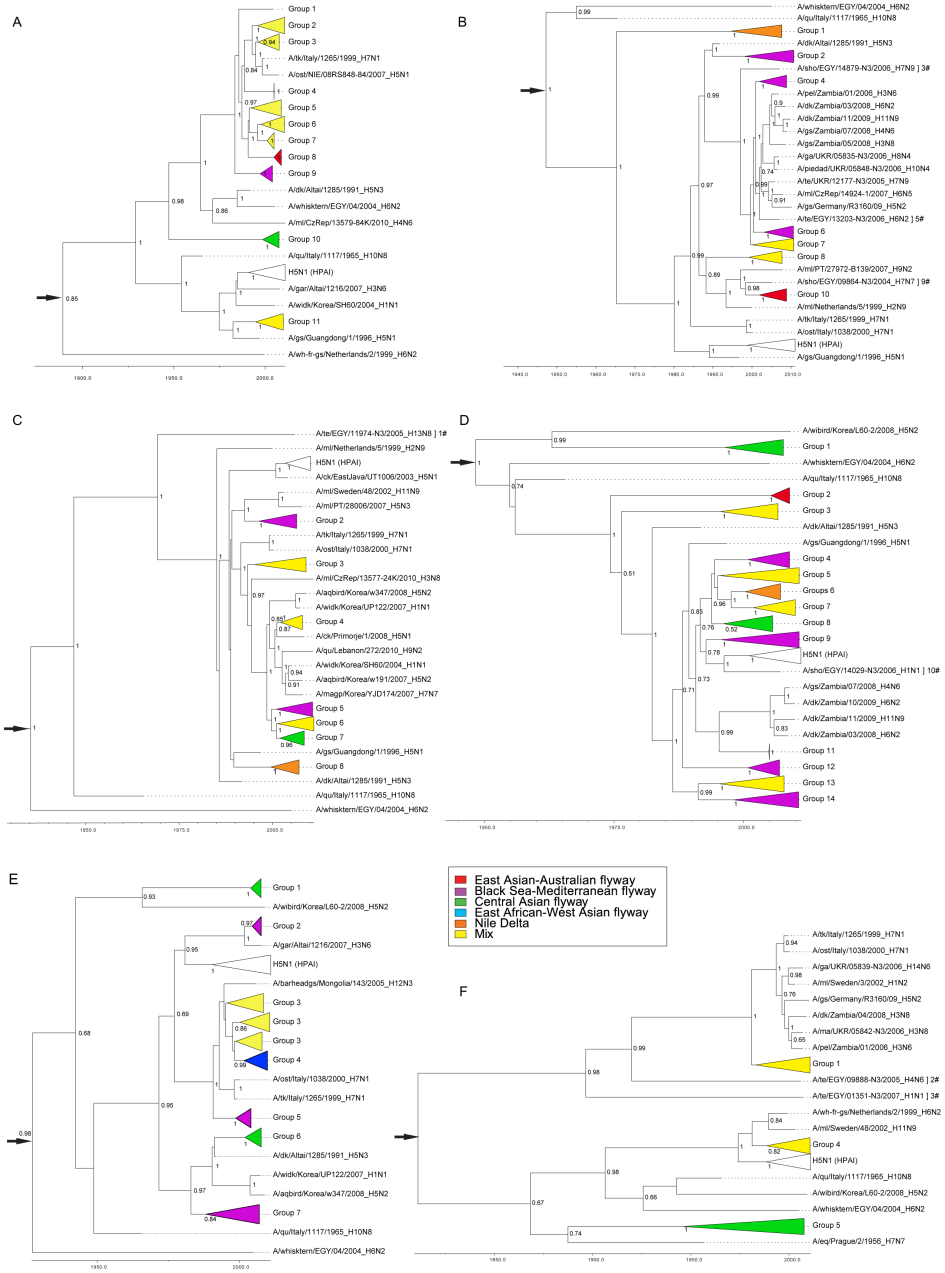
Phylogenies and TMRCA of PB2 (A), PB1 (B), PA (C), NP (D), MP (E) and NS (F) genes inferred with BEAST [26]. Posterior probabilities (>0.7) are shown at each node on the tree and a time scale in years is shown below each tree. Branches containing Egyptian viruses were collapsed around nodes and numbered according to the tree topology from top to bottom. All trees are rooted to the ancestral virus, A/equine/Prague/2/1956 H7N7, and the outlier branch was replaced by a black arrow except NS, which is midpoint rooted. The coloring of collapsed monophyletic groups (key shown in figure inset) corresponds to the predicted flyway (Red for East Asian-Australian flyway, purple for Black Sea-Mediterranean flyway, green for Central Asian flyway, blue for East African-West Asian flyway, orange for Nile Delta and yellow if the group contained 2 or more reference viruses collected from different flyways. Viruses that did not cluster with any other viruses are indicated with (#) behind the number. Abbreviations aq = aquatic, barhead = bar headed, ck = chicken, dk = duck, eq = equine, gar = garganey, gs = goose, magp = magpie, ml = mallard, ost = ostrich, qu = quail, pel = pelican, sho = shoveler, te = teal, tk = turkey, wi = wild, whisk = whiskered, wh-fr-gs = white fronted goose, EGY = Egypt, N3 = NAMRU3, UKR = Ukraine.

To explore the possible genetic exchange between New World and Old World viruses, we analyzed representative (n≥2000) sequences for each gene and inferred phylogenies. Genes from Egyptian viruses were invariably most closely related to European, Asian and African counterparts sharing between 99–96% nucleotide identities indicating that none of these genes originated from the New World (Figure S3). Thus, sequences from North American viruses were excluded from more detailed analysis. The 6 internal genes of Egyptian viruses described here grouped among AIV from many different regions and clustering was independent of subtype, collection date or host species. Most of the Egyptian AIV genes were distinct from highly pathogenic (H5 or H7) viruses from Egypt or elsewhere, and they shared nearest common ancestors with low pathogenic Eurasian AIV. In contrast, the PA genes of three viruses (subtypes H5N2, H7N7, H7N3) shared common ancestors with those of a small group of HPAI H5N1 viruses that did not co-evolve with the majority of H5N1 viruses (data not shown). Notably, all internal gene sequences of A/teal/Egypt/11974-NAMRU3/2005 (H13N8) were most closely related to those of other H16 or H13 viruses and clustered independently from all other viruses (Figure 2, Figure S3). Its nucleotide sequence for each gene was 97% identical to the closest related virus from the same cluster. This is the first isolate of a H13N8 subtype from a duck species and the second duck isolate of any H13 subtype virus (the other is H13N6). Both A/shoveler/Egypt/09782-NAMRU3/2004 and A/shoveler/Egypt/09781-NAMRU3/2004 (H10N7), collected on the same day and location, were identical in their HA gene, and were almost identical in their N7 gene, clustering closest with each other in all internal genes, suggesting sampling of nearly identical viruses (Figures 1, S1, S2). The internal genes of A/teal/Egypt/20431-NAMRU3/2003 (H1N2) were very similar to those of a previously reported A/avian/Egypt/920431/2006 (H9N2) virus (Figure S1).

### Bayesian Analysis and TMRCA Estimates

**Figure 3:**
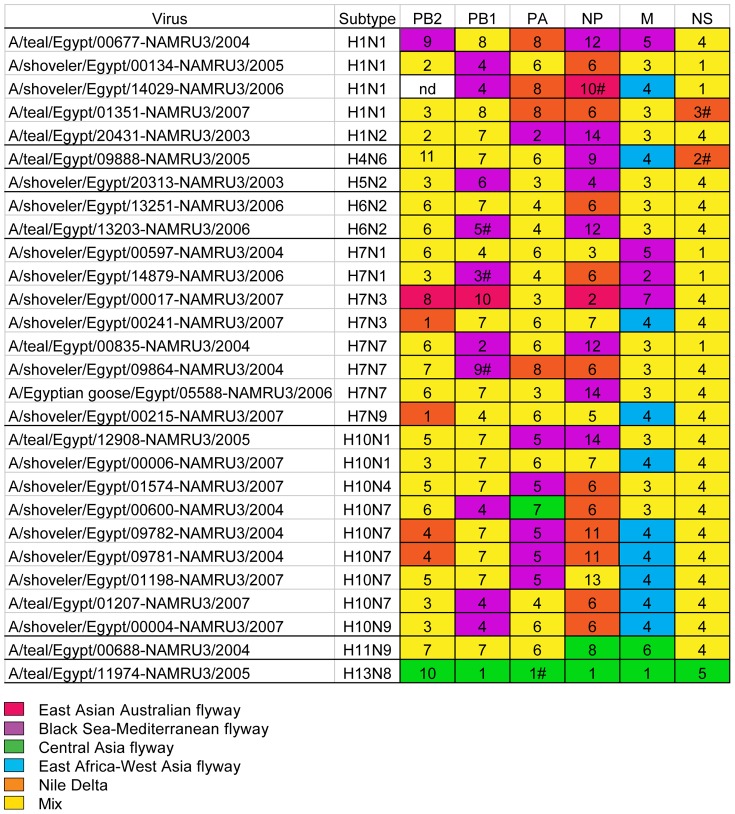
Phylogenetic groups of migratory flyways of viruses from this study. Viruses identified in this study and included in the TMRCA analysis are grouped according to their flyway in the dated phylogenetic tree (Figure 2). Subtypes are also included. Color code is indicated in the figure legend and reflects the inferred flyway in which the Egyptian viruses grouped (red for East Asian-Australian flyway, purple for Black Sea-Mediterranean flyway, green for Central Asian flyway, blue for East African-West Asian flyway, orange for Nile Delta and yellow if the group contained 2 or more reference viruses collected from different flyways). Viruses from this study that did not cluster with any other viruses from Figure 2 are indicated with (#) behind the number and were assigned to flyways that was inferred from large trees (data not shown).

Dated phylogenetic trees using Bayesian analysis were inferred and TMRCA were estimated using small datasets with 92 sequences (including the 28 new isolates described herein). Egyptian viruses from this study were assigned to between 5 (NS) and 14 (NP) distinct phylogenetic groups based on high posterior probability support at a common node (Figures 2 and 3). Most virus sequences clustered with AIV collected from more than one of the four different flyways and in the flyway groupings were neither specific to subtype nor to dates of collection or TMRCA estimates at the common node (Figures 2 and 3).

Unlike all other viruses, the PB2, PB1 and NP genes of A/shoveler/Egypt/00017-NAMRU3/2007 (H7N3) grouped with East Asian sequences indicating genetic transfer from the East Asian gene pool (Figures 2 and 3, Figure S1). The PB2 gene sequences of viruses from Egypt clustered in 11 distinct phylogenetic groups with high statistical confidence as indicated by large posterior probabilities (Figure 2A). A majority of phylogenetic groups (n = 9) contained genes that were associated with 2 or more flyways (BS-MED and EA-WA, Figure 2A). Groups 1 and 4 were comprised of genes clustering only with viruses from the Nile Delta (Figure 2A, Table 3). Group 1 had TMRCA estimates around the year 2004 (HPD 95% 4.72, 9.39) and group 4 had the earliest TMRCA estimate of all the groups in PB2 phylogenies around the year 1988 (HPD 95% 16.72, 29.99). In addition, three viruses clustered in groups with distinct geographic flyways and were segregated from all other Egyptian virus genes (groups 8–10, Figure 2A, Table 2).

**Table 3:** Estimated calendar year (with decimals) of the most recent common ancestor (TMRCA) of phylogenetic groups 1 to 14 described in Figures 2 and 3.

	Gene Segment					
Group	PB2	PB1	PA	NP	M	NS
1	2004.59 (4.77, 9.39)	1994.54 (8.69, 27.44)	1969.23 (29.76, 54.73)	1996.44 (9.02, 21.13)	2003.98 (5.35, 10.9)	1945 (15.75, 151.55)
2	1995.7 (12.08, 20.08)	1998.03 (12.07, 14.74)	1996.28 (12.97, 16.92)	2005.26 (4.56, 7.35)	2004.54 (4.33, 9.86)	1988 (14.15, 36.77)
3	1996.07 (11.87, 19.61)	1997.04 (11.11, 17.73)	1995.16 (16.13, 23.15)	1995.59 (13.11, 18.68)	1990.78 (15.28, 27.74)	2005.72 (5.28, 5.29)
4	1988.64 (16.72, 29.99)	2001.82 (8.15, 10.39)	2002.16 (7.59, 10.27)	2000.8 (8.29, 12.78)	2003.2 (5.98,10.29)	1980.84 (21.14, 46.83)
5	1990.75 (15.07, 27.35)	2002.34 (7.43, 9.98)	2000.94 (8.79, 11.67)	1996.21 (12.02, 18.28)	1998.75 (9.06, 18.2)	2007.03 (3.97, 3.97)
6	1997.73 (10.01, 17.45)	2003.08 (7.22, 9.13)	2000.97 (8.96, 11.41)	2000.34 (8.82, 13.06)	2001.93 (7.11, 12.91)	
7	1995.84 (11.66, 29.2)	2001.51 (8.32, 10.9)	2001.92 (7.81, 10.41)	2001.92 (6.52, 12.13)	1988.31 (15.72, 31.27)	
8	1990.75 (15.07, 27.35)	1999.16 (9.19, 16.02)	1995.5 (9.02, 14.84)	1993.83 (14.29, 20.88)		
9	1997.28 (11.61, 17.73)	1997.09 (10.69, 18.03)		1995.6 (12.63, 18.81)		
10	1998 (7.26, 19.19)			1996.22 (11.8, 18.29)		
11	1994.72 (11.23, 23.62)			1995.28 (10.51, 21.06)		
12				2001.68 (8.02, 11.05)		
13				2003.5 (5.17, 11.52)		
14				1998.09 (9.38, 16.97)		

TMRCA of each internal gene segment group is shown with the 95% HPD in parenthesis.

The PB1 genes clustered in 9 diverse groups with mixed flyways, except for three Egyptian viruses that clustered apart from all other viruses (groups 3, 5, and 9, Figure 2B, Figure 3). Groups 2, 4 and 6 were all related to the Black Sea –Mediterranean and group 10 with East African- West Asian flyway. The TMRCA ranged from 1994 (HDP 95% 8.69, 27.44) for group 1 to 2003 (HDP 95% 7.21, 9.19) for group 6 (Table 2).

In the PA gene, viruses grouped in 8 phylogenetic clusters with group 1 containing only the H13N8 virus, which had an estimated TMRCA around 1969 (HPD 95% 29.76, 54.73). In group 8 there were only viruses from Egypt with a TMRCA around 1995 (Table 2, Figures 2 and 3). Groups 2 and 5 were comprised of viruses that belonged to the Black Sea-Mediterranean flyway. Group 7 viruses clustered with a virus located in the Central Asian flyway and diverged around 2001 (HPD 95% 7.81, 10.41). Group 3 contained viruses that shared common ancestry with 2 HPAI H5N1 viruses (A/whooper swan/Hokkaido/4/2011 and A/grebe/Tyva/2/2010) that had most probably diverged around 2005 (HPD 95% 4.91, 7.1; Table 2). When the same viruses were analyzed in large trees, they clustered with other AIV subtypes (data not shown).

The NP gene sequences clustered in 14 different groups. Group 6, 10 and 11 contained only Egyptian viruses (Figures 2D, 3). Single Egyptian viruses clustered each with distinct viruses from the Central Asian flyway (group 1, and 8), the East Asian-Australian flyway (group 2) and the BS-MED flyway (groups 4, 9, 12, 14) (Figures 2D, 3). TMRCAs ranged from 1993 (HDP 95% 14.29, 20.88, Table 2) in group 8 to the most recent in the year 2005 (HDP 95% 4.56, 7.35) for group 2. A single virus, A/shoveler/Egypt/14029-NAMRU3/2006 (H1N1), clustered closest with HPAI H5N1 viruses of lineage G V X-series Z Z+ and shared a TMRCA around the year 1996 (HPD 95% 11.8, 18.29, Table 2). Large datasets used to generate preliminary trees showed the latter virus shared a node with AIV viruses A/duck/Fujian/12371/2005 (H6N2) and A/chicken/Guangxi/3791/2005 (H5N1). Also, based on the large dataset, group 4 viruses shared ancestors with the Aquatic W lineage of HPAI H5N1 viruses identified in poultry from Southeast Asia (data not shown) [22].

The MP gene phylogenies the Egyptian virus genes were separated into 7 groups (Figures 2E, 3). Five groups contained viruses representing one of the major flyways: Central Asian flyway in groups 1 and 6, the BS-MED flyway in groups 2, 5 and 7 and the EA-WA flyway in group 4 (Figures 2E, 3). The TMRCAs were estimated between the year 1988 (HDP 95% 15.72, 31.27, Table 2) for group 7 and 2004 (HDP 95% 4.33, 9.86) for group 5.

NS gene sequences clustered in 2 alleles with 23 viruses of allele A and 5 viruses of allele B and in 5 separate phylogenetic groups (Figures 2F, 3). Group 5 was comprised of H13 sequences with the common node around the year 1945 (15.75, 151.55), groups 2 and 3 were outlier sequences which did not cluster with any other virus in the small tree (Table 2). The biggest groups were 1 and 4 with at least 2 or more viruses collected from different flyways (Figure 3).

### Molecular Characterization of Amino Acid Sequences

All subtypes differed in the amino acid sequences of the N-terminal side of the cleavage site of the HA but lacked a multiple basic amino acid motif (Table 2). The amino acid residues of HA known to contribute to receptor binding reflected only avian consensus sequences and none of the NA sequences displayed the stalk deletion associated with pathogenicity in HPAI H5N1 viruses [31]. Furthermore, no neuraminidase-inhibitor or adamantane antiviral drug resistance markers were detected [32], [33], [34].

We found some aa residue changes in the internal genes of avian influenza viruses that were described as being involved in enhanced polymerase activity in mammalian cells [35]. All of the Egyptian viruses had the L13P substitution in their PB1 protein but maintained S678 [35]. A/Egyptian goose/Egypt/5588-NAMRU3/2006 (H7N7) had N319K in the NP protein which was also found to increase polymerase activity in H5N1 viruses [35]. This aa replacement was very rare when comparing 2001 aligned avian influenza NP protein sequences. All viruses had amino acids typical of avian influenza viruses in the PB2 protein at residues E627 [36], [37], [38]. Five viruses had the serine at position 66 in their PB1-F2, which has been shown in H5N1 viruses to increase virulence in mammals [39]. All viruses had a full length PB1-F2 (1–87 aa) unlike truncated versions found in the human pdmH1N1 (PB1-F2 encodes 11 amino acids) and human seasonal influenza H1N1 (PB1-F2 encodes 57 amino acids) [40]. Human-influenza associated amino acid residues include PB1-F2 in positions 76, 82, 87 [41], [42], [43]. In our dataset, 22 viruses shared a serine at position 82, one virus had a glycine at position 87, and two viruses had both 82 and 87 residues replaced by aserine (A/shoveler/Egypt/14879-NAMRU3/2006, H7N9; A/teal/Egypt/00677-NAMRU3/2004, H1N1). M1 and M2 protein sequences were typically avian-like. The NS1 proteins of allele B viruses differed in at least 67 amino acids from allele A counterparts. NS1 residues 186 and Phe103/Met106 have been implicated in interaction with anti-IFN capacity [44], [45]. Viruses that belonged to NS allele B in this study had the Phe103Y/Met106 substitution in their NS1, whereas all viruses of allele A had Phe103/Met106. The NS2 of allele B viruses differed by at least 14 amino acids from those of allele A viruses. In the NS2 position, all viruses contained the avian-specific methionine or glutamine at residue 14 and avian-specific serine at position 70 except for 2 viruses with a glycine (A/teal/Egypt/01351-NAMRU3/2007, H1N1 and A/teal/Egypt/11974-NAMRU3/2005, H13N8) [46]. Similar to the phylogenetic divergence, the H13N8 virus differed also in the internal protein amino acid sequence with at least 16 (NP gene), 11 (PB2), 9 (PB1) and 7 (PA) amino acids from any other virus identified in this study.

## Discussion

This study provides a detailed characterization of full genomes of multiple subtypes of low pathogenicity avian influenza A viruses collected from wild birds in Egypt. Notably, 15 different influenza A subtypes, including a rare H13N8 virus subtype, were identified through this multiyear surveillance program. The 28 viruses described were collected during avian influenza surveillance from 2003 through 2007 with all of the mature HA amino acid cleavage sites identified as those typically found in low pathogenicity AI viruses [47]. The six internal genes from all the Egyptian and Ukrainian viruses analyzed in this study were most closely related to genes from other AIV previously detected in the Paleartic ecozone. The internal genes (PB2, PB1, PA, NP, M, and NS) clustered with viruses predominantly of European, African, Central Asian or Far Eastern origin, which is in agreement with the convergence of major migratory bird flyways in the Nile Delta [11], [13], [48]. All sequences showed different clustering patterns for each gene except for H13N8, which always grouped with H13 and H16 viruses (Figure 2; Figure S3). All other Egyptian viruses either formed specific subtype clusters or grouped according to collection date or geographic origin of collection. The HA of the H11 virus (A/teal/Egypt/00688-NAMRU3/2004) grouped interspersed in the tree with other viruses of the same subtype (Figure S1), indicating this could be a sign of sequence gaps because only 68 H11 HA sequences are available from public databases (excluding the New World isolates).

For all genes, except for the NP gene of A/shoveler/Egypt/14029-NAMRU3/2006 (H1N1), virus sequences grouped separately from Egypt HPAI H5N1 viruses. The latter virus shared a most recent common ancestor with HPAI H5N1 viruses circulating around 1996, indicating a common evolutionary pathway in a wild bird reservoir. Most of the viruses showed a high similarity in their nucleotide sequence to other low pathogenic viruses from the three flyways. However, some genes formed independent groups with viruses sequenced only for this study suggesting these viruses were enzootic in the region or represented sampling bias in the birds surveyed. Other genes did not cluster with any other virus present indicating significant sequence gaps.

Previously described genotypes of HPAI H5N1 viruses were applied in phylogenetic analyses but were not specifically used to define specific clustering for LPAI viruses described in this study. For the PA gene, some H5N1 HPAI viruses formed clusters with LPAI viruses and were separate from all other known H5N1 viruses. These 3 H5N1 viruses belonged to clade 2.3.2.1 (based on WHO/OIE/FAO HA clade nomenclature) [49]. These viruses were found both in domestic poultry and wild birds indicated a possible transmission route between the different hosts. According to the genotyping for H5N1 viruses, the PA sequence of virus A/chicken/Primorje/1/2008 belongs to the Aquatic V lineage usually found in aquatic birds. This may be a sign of a previous exchange of PA gene segments between low pathogenic influenza in wild birds or poultry and HPAI viruses of subtype H5N1. However, the lack of LPAI genes found in circulating H5N1 viruses in Egyptian poultry indicates there are few opportunities for viruses of wild bird-origin to reassort with viruses of poultry-origin [50].

Most of the densely populated areas of the country are on or around major water reservoirs, such as the Nile Delta in the northeast, the Nile River in central and Upper Egypt, and in the southeastern part of the country around Lake Nasser and the Aswan dam region. Low pathogenic viruses have not been identified from domestic poultry in Northeast Africa and might be under-reported due to sequence gaps unlike in Sub-Saharan and South African regions where wild bird surveillance was reported [51]. Additionally, the low pathogenic viruses cause only mild symptoms in migratory birds and escape surveillance targeted towards sick or dead birds [52].

The seasonal migration patterns facilitate contact and possible transmission of influenza viruses to poultry especially for *Anseriformes* such as mallard, teal and shoveler. Mallard species migrate to their winter, non-breeding sites located in the North of Egypt including the Nile Delta region [53]. Other flyways may contribute to diversity of low pathogenic sequences in Egypt, such as the Central Asian, the East Asian- Australian, and East Atlantic flyways, where breeding regions of *Anseriformes* species reside (Asia-Pacific migratory waterbird conservation strategy: 2001–2005 2001; Stroud 2004). Two of the major migratory flyways overlap in Egypt, including the Black-Sea-Mediterranean and the East African-West Asian flyway [13], [48], which was a likely contributing factor to the extensive genetic diversity described in this study. Unlike all other viruses, A/shoveler/Egypt/00017-NAMRU/2007 (H7N3) clustered closely with Southeast Asian viruses in PB2, PB1 and NP gene sequences. Furthermore, for several genes and viruses, close grouping with viruses from the Central Asian flyway was observed. Both indicate that not only genes derived from AIV of the BS-MED and EA-WA flyways contribute to the gene pool in Egypt’s LPAI viruses, but also genes from the Central Asian and Southeast Asia flyways. The large gene pool of the internal genes has a low divergence when compared to the external genes HA and NA and is characteristic for low pathogenic AIV. Thus, the identification and significance of possible reassortment among these viruses were difficult to establish [54]. Migratory birds carrying influenza viruses travel long distances in large groups and could intermingle with local bird populations transmitting influenza viruses to local wild birds and free range poultry [55]. Our data support these findings with genes sequences that evolved independently and grouped with viruses of diverse origins and lacking a defined association by subtype, origin or date of collection. Furthermore, the phylogenetic groups that we defined for each gene shared a most common recent ancestor dating within 5 to 10 years prior to the virus sample collection date (2003–2007), except for H13N8 virus genes. This would be consistent with the notion that LPAI viruses undergo exchange of internal genes regularly. We observed low sequence diversity in sequences from internal genes and interspersed clustering, unlike that observed for highly pathogenic viruses circulating in poultry that have diversified into different genotypes [22].

For the first time we analyzed full genome sequences of multiple subtypes not reported previously in Africa, including H1N1, H7N1, H10N4 and H10N7. In addition, this is the first subtype H13N8 isolated from a duck (*Anas crecca*; Common teal, Eurasian teal) and is only the third full genome of this subtype available in public databases. Remarkably, the H13N8 virus clustered separately in all genes and differed substantially compared to any other subtype except for H16 viruses. The high diversity may indicate a separate gene pool for these two subtypes or gaps in surveillance. We found that the H13N8 sequences were at least 7 (PA) to 16 (NP) amino acids different from any of the other viruses identified here. The high divergence in the NP protein could indicate a possible link for this species restriction, as nucleoprotein sequences were species specific in phylogenetic comparisons. The H13 subtypes are commonly found in shorebirds (Charadriiformes) [56] and rarely reported from *Anseriformes* in Siberia/Japan [57], and Alaska [58]. It was hypothesized previously that the predominant isolation of H13 and H16 viruses from gull species confirms the common notion that these viruses belong to the influenza A virus “gull lineage” [59]. H13 and H16 viruses are genetically distinct from viruses from other hosts and seem to have adapted to replication in gull hosts in particular [60], [61]. Extensive studies are needed to determine whether the duck isolate from this study was a transient infection acquired from a shorebird or indeed a virus adapted to ducks.

In conclusion, here we show for the first time LPAI virus sequences identified in Egypt from 2003 to 2007 illustrating the importance of the Nile Delta as a funnel for a large diversity of influenza A viruses. Internal gene sequences were closely related to Eurasian, African and/or Middle East-origin viruses. Using Bayesian phylogenies with TMRCA analyses, the internal genes, in general, should be grouped according to specific migratory bird flyways indicating the Nile Delta serves as a source for a widely distributed influenza A virus gene pool. Most recent common ancestors identified were 5 to 10 years before the collection date of the viruses, except for H13 viruses. All viruses showed a low sequence variation, except for the H13N8 subtype, which clustered separately from all other viruses and was found for the first time in a duck species.

## Supporting Information

Figure S1 **Phylogenetic trees of HA genes analyzed using large datasets of publicly available sequences and LPAI viruses sequenced from Egypt (boldface).** The eight trees are labeled with their HA subtype H1 (A), H4 (B), H5 (C), H6 (D), H7 (E), H10 (F), H11 (G), H13 (H). Separate branches were collapsed and labeled according to their host and location if they did not contain any virus from this study (e.g. North American Avian). NJ trees were calculated with Mega4 [25] and Kimura 2-parameter distance model. The subtypes are shown behind virus names; in (D) all viruses were of subtype H6N2. In H7 (E) high pathogenicity viruses are indicated by+following the virus names.
(PDF)

Figure S2 **Phylogenetic trees of NA genes analyzed using large datasets of publicly available sequences and LPAI viruses sequenced from Egypt (boldface).** The eight trees are labeled with their NA subtype N1 (A), N2 (B), N3 (C), N4 (D), N6 (E), N7 (F), N8 (G), N9 (H). Separate branches were collapsed and labeled according to their host and location if they did not contain any virus from this study (e.g. North American Avian). NJ trees were calculated with Mega4 [25] and Kimura 2-parameter distance with subtypes shown behind virus names.
(PDF)

Figure S3 **Reference viruses to phylogenetic groups described in this study by group (** **Figures 2** **and** **3** **) and gene segment.** Kimura 2-parameter distances were calculated from bigger alignments in Mega4 (Tamura et al. 2007). Color codes reflect flyways the reference viruses belong to. Abbreviations aq = aquatic, av = avian, barhead = bar headed, ck = chicken, dk = duck, eggs = Egyptian goose, eq = equine, gar = garganey,gl = gull, gs = goose, magp = magpie, ml = mallard, ost = ostrich, qu = quail, pel = pelican, sho = shoveler, te = teal, tk = turkey, wi = wild, whisk = whiskered, wh-fr-gs = white fronted goose, EGY = Egypt, GD = Guangdong, N3 = NAMRU3, PT = Portugal, Rep = Republic, UKR = Ukraine.
(PDF)

Table S1 **Primer sequences used in the multiplex subtype detection assay.**
(XLSX)
