# Supplementary figures and images for: A High Diversity of Eurasian Lineage Low Pathogenicity Avian Influenza A Viruses Circulate among Wild Birds Sampled in Egypt
Source: PLoS One. 2013 Jul 12;8(7):e68522. doi: 10.1371/journal.pone.0068522 (PMC3710070; doi:10.1371/journal.pone.0068522)

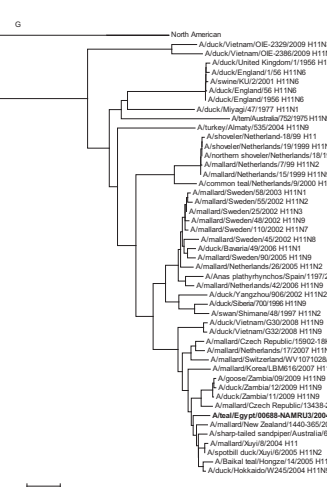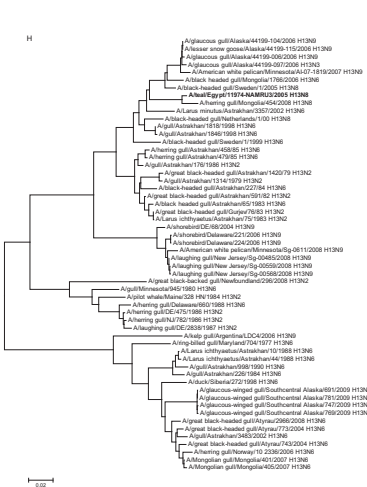

Supplement: Figure S1 — Phylogenetic trees of HA genes analyzed using large datasets of publicly available sequences and LPAI viruses sequenced from Egypt (boldface). The eight trees are labeled with their HA subtype H1 (A), H4 (B), H5 (C), H6 (D), H7 (E), H10 (F), H11 (G), H13 (H). Separate branches were collapsed and labeled according to their host and location if they did not contain any virus from this study (e.g. North American Avian). NJ trees were calculated with Mega4 [25] and Kimura 2-parameter distance model. The subtypes are shown behind virus names; in (D) all viruses were of subtype H6N2. In H7 (E) high pathogenicity viruses are indicated by+following the virus names. (PDF) [file pone.0068522.s001.pdf]
