# Supplementary material for: A High Diversity of Eurasian Lineage Low Pathogenicity Avian Influenza A Viruses Circulate among Wild Birds Sampled in Egypt
Source: PLoS One. 2013 Jul 12;8(7):e68522. doi: 10.1371/journal.pone.0068522 (PMC3710070; doi:10.1371/journal.pone.0068522)
Supplement: Figure S3 — Reference viruses to phylogenetic groups described in this study by group ( Figures 2 and 3 ) and gene segment. Kimura 2-parameter distances were calculated from bigger alignments in Mega4 (Tamura et al. 2007). Color codes reflect flyways the reference viruses belong to. Abbreviations aq = aquatic, av = avian, barhead = bar headed, ck = chicken, dk = duck, eggs = Egyptian goose, eq = equine, gar = garganey,gl = gull, gs = goose, magp = magpie, ml = mallard, ost = ostrich, qu = quail, pel = pelican, sho = shoveler, te = teal, tk = turkey, wi = wild, whisk = whiskered, wh-fr-gs = white fronted goose, EGY = Egypt, GD = Guangdong, N3 = NAMRU3, PT = Portugal, Rep = Republic, UKR = Ukraine. (PDF) [file pone.0068522.s003.pdf]

**Group**	**1**	**2**	**3**
**Gene**			
**PB2**	Egypt (2 viruses)	A/ml/Sweden/3/2002 (H1N2)	A/Eggs/SouthAfrica/AI1448/2007 (H1N8)
**PB1**	A/Mongolian gl/Mongolia/401/2007 (H13N6)	A/ml/Sweden/48/2002 (H11N9)	A/sho/Egypt/14879-NAMRU3/2006 (H7N9)
**PA**	A/tl/Egypt/11974-NAMRU3/2005 (H13N8)	A/av/Egypt/920431/2006 (H9N2)	A/barheadgs/Mongolia/143/2005 (H12N3)
**NP**	A/Mongolian gl/Mongolia/401/2007 (H13N6)	A/aqbird/Korea/w347/2008 (H5N2)	A/ost/Italy/1038/2000 (H7N1)
**M**	A/Mongolian gl/Mongolia/401/2007 (H13N6)	A/ml/PT/28006/2007 (H5N3)	A/ml/Netherlands/5/1999 (H2N9)
**NS**	A/Mongolian gl/Mongolia/401/2007 (H13N6)	A/ml/Netherlands/5/1999 (H2N9)	A/tl/Egypt/09888-NAMRU3/2005 (H4N6)
**Group**	**4**	**5**	**6**
**Gene**			
**PB2**	A/sho/Egypt/09781-NAMRU3/2004 (H10N7)	A/ml/Sweden/48/2002 (H11N9)	A/aqbird/India/NIV-17095/2007 (H11N1)
**PB1**	A/ml/PT/28006/2007 (H5N3)	A/tl/Egypt/13203-NAMRU3/2006 (H6N2)	A/qu/Lebanon/272/2010 (H9N2)
**PA**	A/pel/Zambia/01/2006 (H3N6)	A/ml/CzechRep/13579-84K/2010 (H4N6)	A/gb/Tyva/2/2010 (H5N1)
**NP**	A/ml/PT/28006/2007 (H5N3)	A/wh-fr-gs/Netherlands/2/1999 (H6N2)	Egypt
**M**	A/gs/Zambia/07/2008 (H4N6)	A/ml/Sweden/3/2002 (H1N2)	A/gar/Altai/1213/2007 (H5N2)
**NS**	A/dk/Altai/1285/1991 (H5N3)	A/tl/Egypt/01351-NAMRU3/2007 (H1N1)	
**Group**	**7**	**8**	**9**
**Gene**			
**PB2**	Egypt (2 viruses)	A/wibird/Korea/L60-2/2008 (H5N2)	A/ml/Netherlands/5/1999 (H2N9)
**PB1**	A/Eggs/SouthAfrica/AI1448/2007 (H1N8)	A/barheadgs/Mongolia/143/2005 (H12N3)	A/widk/Korea/SH60/2004 (H1N1)
**PA**	A/gar/Altai/1216/2007 (H3N6)	A/gs/GD/1/1996 (H5N1)	
**NP**	A/gar/Altai/1216/2007 (H3N6)	A/barheadgs/Mongolia/143/2005 (H12N3)	A/ml/CzechRep/13577-24K/2010 (H3N8)
**M**	A/wh-fr-gs/Netherlands/2/1999 (H6N2)		
**Group**	**10**	**11**	**12**
**Gene**			
**PB2**	A/Mongolian gl/Mongolia/401/2007 (H13N6)	A/pel/Zambia/01/2006 (H3N6)	
**NP**	A/sho/Egypt/14029-NAMRU3/2006 (H1N1)	Egypt (2 viruses)	A/ml/Sweden/48/2002 (H11N9)
**Group**	**13**	**14**	
**Gene**			
**NP**	A/ml/Sweden/3/2002 (H1N2)	A/av/Egypt/920431/2006 (H9N2)	

- East Asian Australian flyway
- Black Sea-Mediterranean flyway
- Central Asia flyway
- East Africa-West Asia flyway
- Nile Delta
