# Supplementary material for: A High Diversity of Eurasian Lineage Low Pathogenicity Avian Influenza A Viruses Circulate among Wild Birds Sampled in Egypt
Source: PLoS One. 2013 Jul 12;8(7):e68522. doi: 10.1371/journal.pone.0068522 (PMC3710070; doi:10.1371/journal.pone.0068522)
Supplement: Figure S2 — Phylogenetic trees of NA genes analyzed using large datasets of publicly available sequences and LPAI viruses sequenced from Egypt (boldface). The eight trees are labeled with their NA subtype N1 (A), N2 (B), N3 (C), N4 (D), N6 (E), N7 (F), N8 (G), N9 (H). Separate branches were collapsed and labeled according to their host and location if they did not contain any virus from this study (e.g. North American Avian). NJ trees were calculated with Mega4 [25] and Kimura 2-parameter distance with subtypes shown behind virus names. (PDF) [file pone.0068522.s002.pdf]

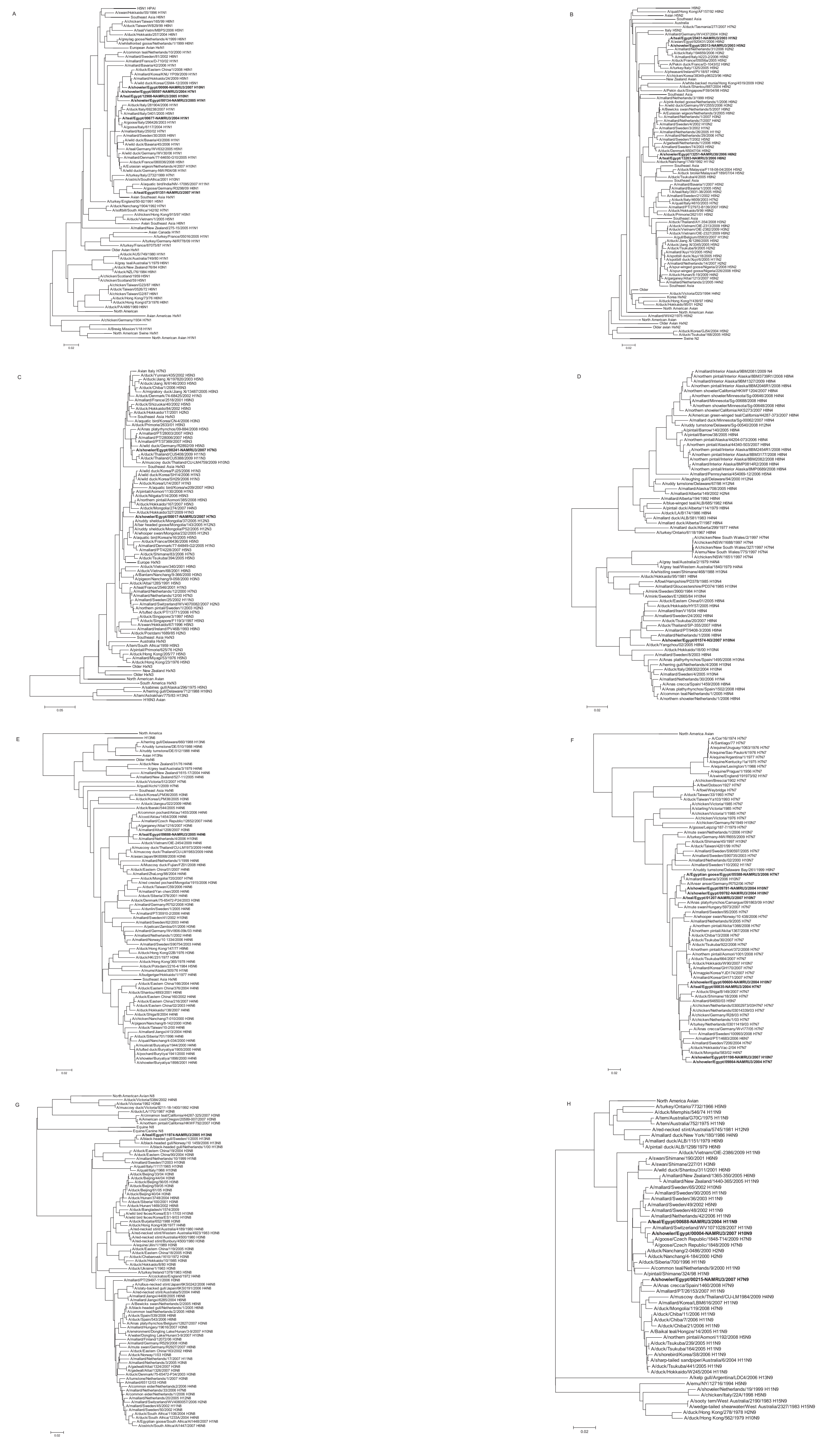
A
B
C
D
E
F
G
H
